# The Importance of Digging into the Genetics of *SMN* Genes in the Therapeutic Scenario of Spinal Muscular Atrophy

**DOI:** 10.3390/ijms22169029

**Published:** 2021-08-21

**Authors:** Mar Costa-Roger, Laura Blasco-Pérez, Ivon Cuscó, Eduardo F. Tizzano

**Affiliations:** 1 Medicine Genetics Group, Vall d’Hebron Research Institute (VHIR), 08035 Barcelona, Spain; mar.costa@vhir.org (M.C.-R.); laura.blasco@vhir.org (L.B.-P.); icusco@vhebron.net (I.C.); 2 Department of Clinical and Molecular Genetics, Hospital Vall d’Hebron, 08035 Barcelona, Spain; 3 Centro de Investigación Biomédica en Red de Enfermedades Raras (CIBERER), 08035 Barcelona, Spain

**Keywords:** spinal muscular atrophy, survival motor neuron 1, survival motor neuron 2, genotype-phenotype correlations, variants, hybrid structure

## Abstract

After 26 years of discovery of the determinant survival motor neuron 1 and the modifier survival motor neuron 2 genes (*SMN1* and *SMN2*, respectively), three SMN-dependent specific therapies are already approved by FDA and EMA and, as a consequence, worldwide SMA patients are currently under clinical investigation and treatment. Bi-allelic pathogenic variants (mostly deletions) in *SMN1* should be detected in SMA patients to confirm the disease. Determination of *SMN2* copy number has been historically employed to correlate with the phenotype, predict disease evolution, stratify patients for clinical trials and to define those eligible for treatment. In view that discordant genotype-phenotype correlations are present in SMA, besides technical issues with detection of *SMN2* copy number, we have hypothesized that copy number determination is only the tip of the iceberg and that more deepen studies of variants, sequencing and structures of the *SMN2* genes are necessary for a better understanding of the disease as well as to investigate possible influences in treatment responses. Here, we highlight the importance of a comprehensive approach of *SMN1* and *SMN2* genetics with the perspective to apply for better prediction of SMA in positive neonatal screening cases and early diagnosis to start treatments.

## 1. SMA Is a Disease of Two Genes, a Determinant *SMN1* and a Modifier *SMN2*

Spinal muscular atrophy (SMA) is a severe neuromuscular disease characterized by progressive proximal muscle weakness and atrophy as a result of alpha neuron degeneration and irreversible loss in the spinal cord anterior horn [1]. Overall estimated incidence is 1 in 11,000 live births with a carrier frequency around 1/54 [2]. Despite SMA clinically manifests as a continuum, based on age of onset, achieved motor milestones and clinical severity, SMA patients are divided into type 0-IV ranging from very severe congenital forms with short life expectancy due to respiratory failure at birth to adult-onset patients maintaining the ability to walk [3,4]. 

At the molecular level, SMA is an autosomal recessive disorder caused by loss of survival motor neuron 1 (*SMN1*, OMIM #600354) gene in the 5q13 locus [5]. Around 95% of cases are explained by homozygous deletion or gene conversion, while a minority of patients are compound heterozygous including intragenic pathogenic variants and deletion of one *SMN1* allele [6]. 

Besides *SMN1* gene there is *SMN2* (OMIM #601627), an almost equal centromeric paralog gene. Both *SMN* genes have an identical genomic organization consisting of nine exons interrupted by eight introns, which fits with the recent duplication of the *SMN1* gene, explaining why *SMN2* is only present in humans [5,7,8]. The exact structure of the SMA region remains unclear and initially *SMN1* and *SMN2* were described to be in opposite directions (head-to-head) [5], but more recently, evidence supports that the two genes are oriented in the same direction [9]. More studies are needed to determine which structure is correct or if both orientations are present in the population. 

**Figure 1:**
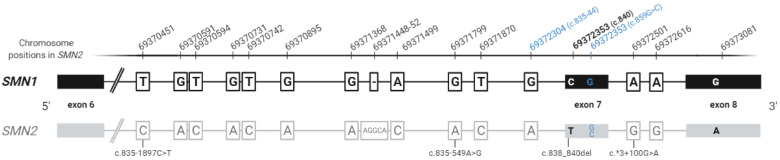
Schematic view of paralogous sequence variants (PSVs) between *SMN1* and *SMN2*. PSVs are represented in black in *SMN1* and in grey in *SMN2* and validated positive modifiers (c.835-44A>G and c.859G>C) in blue. The PSV that changes the splicing pattern of *SMN2* (c.840C>T) is highlighted in bold. Putative *SMN2* modifiers are indicated below the *SMN2* scheme. Note that c.859G>C is not a PSV, but a rare variant present only in *SMN2* gene. Chromosome positions in *SMN2* refer to hg19. Based on reference [10].

Given that *SMN2* was originated from *SMN1,* their sequences only differ in 16 paralogous sequence variants (PSVs), which represent a total of 20 different nucleotides between both genes (15 SNVs and 1 indel) [10] as represented in Figure 1. The PSV c.840C>T, located in exon 7, causes exon skipping in the majority of *SMN2* pre-mRNA transcripts resulting in a truncated, nonfunctional and rapidly degraded protein that is not able to oligomerize (SMN-∆7) [11] and can only produce the complete functional protein in 10–15% of cases [12,13,14]. Conversely, *SMN1* gene produces virtually full-length mRNA transcripts encoding the normal SMN protein. Bi-allelic alteration of *SMN1* is the rule to confirm SMA, however *SMN2* copies varying from 1 to 5 are present in all patients, as absence of both genes has never been reported in humans. An inverse correlation between *SMN2* copy number (*SMN2*_CN) and disease severity is currently accepted, being the number of *SMN2* copies the main modifier of the SMA phenotype (see Section 2) [5,15]. 

The SMA genomic region is highly polymorphic and dynamic, which is prone to unequal rearrangements leading to deletions, duplications or gene conversions [7]. In fact, the presence of *SMN1* or *SMN2* genes lacking exons 7 and 8 (*SMN1/2*∆7-8) has been reported in the general population, with the breakpoint described in intron 6 [16,17]. The frequency of this variant varies greatly among populations including 15–21% in non-Finish Europeans, 7–11.5% in Americans and Finnish European individuals and 0.3–3% in Asian and African populations [17,18]. Several studies observed a strong inverse correlation between this partial deletion and *SMN2*_CN suggesting that the *SMN1/2*∆7-8 variant is mainly derived from *SMN2* deletion events [17,19].

Apart from partial deletions of *SMN* genes, other structural variants have been characterized such as hybrid *SMN1*-*SMN2* genes. Around 5–10% of SMA patients show homozygous deletions of exon 7, but not of exon 8 of *SMN1* explained by the presence of hybrid genes [6,20,21]. This phenomenon could result from intrachromosomal deletions or more likely from gene conversion events in which part of the *SMN2* gene is fused to *SMN1* [20,22]. Although there is still some debate, many studies found that hybrid *SMN* genes appeared to be associated with a milder phenotype, mainly present in SMA type II and III patients [20,21,23]. 

Copy number of *SMN1* gene have also been described in the general population with differences across various ethnicities, with a higher average of *SMN1* copies in African American population [24]. In fact, 54.7% of Africans carry three or more *SMN1* copies according to a recent study [18]. In this context, the frequency of silent 2/0 carriers (individuals with two *SMN1* copies in cis) is also higher as it is directly related to the frequencies of *SMN1* deletions and duplications [25,26]. The detection of 2/0 carriers is challenging given the difficulty to differentiate them from 1/1 non-carriers. Interestingly, two *SMN1* variants have been associated with silent carriers in the Ashkenazi Jewish population, including c.*3+80T>G and c.*211_*212del, which ultimately modify the SMA carrier risk being useful to detect around 20% of these special carriers [25,27]. However, it must be taken into account that there are several 2/0 cases without these variants [27]. 

Therefore, an accurately deep characterization of the SMA region is relevant not only for the detection of *SMN1* and *SMN2* copy number, but also for the different structural variants described. There are complex biological features of the SMN region that hinder the analysis of these genes, including the high homology between both genes, the multiple *SMN2* copies, the presence of partial deletions and hybrid structures and the effect of unknown intronic variants (Table 1). Technical limitations may include difficulties to ascribe a variant to *SMN1* or *SMN2*, quantitation of *SMN2* may not be always straightforward because of sample or methodological problems and structural changes are not usually detected with routine methods. 

## 2. The Known Validated Genotypes

Determination of *SMN2*_CN is a useful prognostic tool in order to establish accurate genotype-phenotype correlations, predict disease course and determine appropriate SMA patients for treatment [15]. Calucho et al. (2018) compiled a total of 3459 SMA patients and established quantitative *SMN2* correlations to predict disease evolution. Concretely, the higher number of *SMN2* copies, the milder the SMA phenotype, as most patients comply with the following rule: SMA type I patients had 2 *SMN2* genes, type II had 3 *SMN2*, type III had 3 or 4 *SMN2* copies and type IV patients had 4 *SMN2* genes [15]. Later on, Ruhno et al. (2019) proposed a model to classify patients based on their *SMN2* dosage, including concordant patients with an expected *SMN2*_CN for their disease severity, and discordant patients with either a milder or more severe phenotype. This model only differs from Calucho’s correlations in type III patients, since it establishes the expected *SMN2*_CN to be 4 instead of 3 and 4 indistinctly [9]. In the recent literature, the correlation described by Calucho et al. (2018) is mainly maintained [28,29,30,31,32], although some exceptions can be found. Interestingly, the proportion of type I patients with 3 *SMN2* genes was increased in some cohorts, reaching 57% among SMA I patients, while Calucho’s compilation reported only 23% [33,34]. In contrast, the cohort described by Sun et al. (2020) seems to have, in global numbers, a better-than-expected phenotype, as the majority of SMA type II patients presented 2 *SMN2* genes, type III patients had 2 or 3 *SMN2* copies and more than half of SMA type IV had only 3 *SMN2* genes [35]. Despite these differences in the literature, all studies agree that the higher the *SMN2*_CN, the less severe the SMA phenotype. This widely described correlation is coherent, since the higher number of *SMN2* copies, the higher amounts of SMN functional protein produced compensating the lack of *SMN1* gene and explaining the better prognosis of the patients.

Nevertheless, this correlation is not absolute and some discordant cases based on this rule are found, which can be further subdivided in “better-than-expected” or “worse-than-expected” patients [15]. In some of these individuals, apart from the *SMN2*_CN, different variants have been reported to modify the SMA phenotype, which can help inform prognostic outcomes. Two positive modifiers in *SMN2* gene have been described, both associated with a milder phenotype [36,37,38]. The first modifier described was the variant c.859G>C (p.Gly287Arg) located in exon 7 of *SMN2*. It creates a novel exonic splicing enhancer site (SF2/ASF motif) predicted by ESEfinder 3.0 [36]. Through SMN splicing assays, it has been demonstrated to significantly increase *SMN2* exon 7 inclusion in vitro from 40–50% to 70% and subsequently the amount of full-length *SMN* transcript [36,37]. There is an additive effect of this variant, as the greater number of *SMN2* copies with c.859G>C, the better the phenotype of the patient. In addition, it has been postulated that this allele has originated from a common ancestor by haplotype analysis [39]. The second variant classified as a positive modifier is the variant c.835-44A>G, located in intron 6, and it is one of the 16 PSVs described between *SMN1* and *SMN2* [10,40]. Wu et al. (2017) demonstrated that this transition decreases the affinity of the RNA-binding protein HuR, which acts as a splicing repressor, increasing in ~20% the *SMN2* exon 7 inclusion [38]. Despite the rare frequency of at least c.859G>C variant (0.8%, 11/1345) [41], a recent SMA practical guideline recommends the evaluation of both variants in discordant SMA patients presenting a better-than-expected phenotype [42].

Other known modifier variants that could explain discordant cases based on *SMN2*_CN include *SMN1* intragenic variants. More than 80 pathogenic variants have been described in *SMN1* gene in compound heterozygous individuals, mainly located in the Tudor and C-terminal domains [43]. In general terms, in the Tudor domain, missense mutations appear to be associated with a more severe SMA phenotype, whereas in the context of frameshift and nonsense variants may be more dependent on the *SMN2* copies [44,45]. For instance, c.275G>C (p.Trp92Ser) variant has been reported in severe SMA type I patients with 3 *SMN2* copies and a reduced interaction with SMN target proteins has been shown using a protein binding assay [46,47,48]. On the other hand, mutations in the C-terminal domain appear mostly related to a worse-than-expected phenotype as is the case of the variant c.770_780dup (p.Leu261Alafs*5) [21,31,49]. In our experience, compound heterozygous patients with this pathogenic variant and the *SMN1* deletion carrying only one *SMN2* copy had congenital type 0 disease [50], whereas patients with two or three *SMN2* copies had type I or II disease, respectively [21]. 

In addition, some missense variants in exon 1 of *SMN1* are associated with a milder phenotype [44]. Two recurrent variants are c.5C>T (p.Ala2Val) and c.5C>G (p.Ala2Gly), which are considered hypomorphic alleles identified in SMA type III patients [51,52]. In fact, it has been shown in a SMA mouse model that the change p.Ala2Gly does not produce total loss of protein function [53] and no significant decrease of full-length *SMN1* transcripts [43]. All these cases highlight the relevance of (1) performing additional functional studies to further characterize *SMN1* pathogenic variants, both including novel and previously described variants, and (2) further characterize the *SMN2* copies in those patients to better explain their phenotypes. 

## 3. The Unknown or Yet Non-Validated Genotypes

Aside from the previously described *SMN2* modifiers (c.859G>C and c.835-44A>G), other variants in this gene have been proposed to modify the SMA phenotype, although functional studies to demonstrate an effect in the SMN protein have not been performed or more cases have not yet been reported [9,38,54]. For instance, variants c.835-1897C>T and c.835-549A>G in intron 6 of *SMN2* and variant c.*3+100G>A in intron 7, later classified as a PSV [10], have been associated with a better-than-expected phenotype (Figure 1) [9,38]. Furthermore, a recent study suggested that variants c.81+45C>T in intron 1 of *SMN2* and c.838_840del in exon 7 were related to a more severe SMA phenotype. This work also identified a novel variant (c.-14C>T) in the promoter region of the *SMN2* gene in an SMA type I patient with apparently four *SMN2* copies indicating a possible association with a worse-than-expected phenotype [54]. In addition, variants in the *SMN1* promoter, which have been associated with non-functional *SMN1* alleles, have also been reported, even though they are infrequent findings [45]. It is important to bear in mind that variants located in deep intronic regions would not be detected through MLPA or exome sequencing, highlighting the importance of recently developed strategies for the entire sequencing of *SMN1* or *SMN2* genes [10,18]. Another cause of discordance could be a clinical misclassification of the patient or an inaccurate *SMN2*_CN determination. On the one hand, the lack of clinical information or the modification of the patient’s phenotype due to natural history or evolving trajectory because of current treatments could lead to reassign the SMA type, thus generating discordance with its *SMN2*_CN [8]. On the other hand, many factors can affect the determination of *SMN2* dosage, giving an inaccurate result. Although MLPA is considered the gold standard technique to detect the number of *SMN2* copies in SMA patients, real-time PCR (RT-PCR) and droplet digital PCR (ddPCR) are also commonly used. In MLPA and RT-PCR based on SYBR Green, DNA quality is crucial to achieve reliable results, and moreover, both methods can be affected by variants in the target region of primers or probes, leading to a misinterpretation of the *SMN2*_CN. In addition, control samples or references are needed in all the approaches since they are based on indirect quantification, and therefore the right choice of these controls is decisive to establish the correct *SMN2*_CN [42]. This fact is reflected in the work of Schorling et al. (2019), in which 20 SMA patients were retested for their *SMN2* dosage using new DNA samples and 45% of the results were discrepant in comparison with the initial ones [55]. Retesting of cohorts with discrepancies (i.e., [33,34,35]) would be interesting to confirm if some of the results are due to problems with *SMN2*_CN determination or clinical misclassification of patients. In fact, a guideline is proposed to manage the discordant situations that are present in SMA patients [42]. 

**Figure 2:**
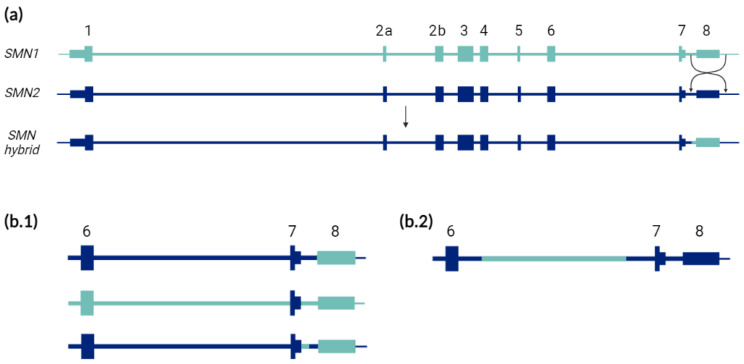
Schematic representation of different *SMN2/SMN1* hybrid structures reported in SMA patients. (**a**) Mechanism of formation of the classical *SMN2/SMN1* hybrid structure detectable through MLPA and other routine techniques and usually defined as “homozygous deletion of only exon 7 of the *SMN1* gene”. (**b.1**) Previously reported *SMN2/SMN1* hybrid structures indistinguishable from the classical one using MLPA and other routine techniques [20,43,51]. (**b.2**) Previously reported *SMN2/SMN1* hybrid structure undetectable through MLPA and other routine techniques [10]. The hybrid structures from (**b**) are detectable by sequencing of the entire *SMN* genes. The different PSV sequences in each structure may determine the expression and functionality of each of the hybrid genes. Light blue represents *SMN1*, and dark blue *SMN2*.

There are also some other genetic factors to consider that remain undetectable with routine techniques. First, different *SMN1*-*SMN2* hybrid structures have been described, but using the available MLPAs only those hybrids formed by exons 1 to 7 of *SMN2* and exon 8 of *SMN1* (more common) or vice versa are detectable (see Figure 2). Blasco-Pérez et al. (2021) described two SMA patients with hybrid structures consisting of the entire *SMN2* gene except for a region of intron 6, corresponding to *SMN1* [10]. This hybrid is undetectable using MLPA as this technique has only specific probes of *SMN1* and *SMN2* in exons 7 and 8 based on the exonic PSVs. Similarly, other uncommon hybrids (such as those reported by Cusco et al. (2001) [20], Qu et al. (2016) [43], Kubo et al. (2015) [51] or Blasco-Pérez et al. (2021) [10]) or partial deletions of *SMN2* copies, would be detectable by the complete sequencing of *SMN* genes or by the study of PSVs of the region [10,18]. Second, besides the common polymorphic *SMN1/2*∆7-8, other partial deletions have been described, some of which are difficult to elucidate if they are located in *SMN1* or *SMN2* genes, further contributing to the complexity of the analysis [18,56]. Third, it has been described that *SMN2* can be hypermethylated, resulting in a partial inactivation of the gene expression, which translates into a worse-than-expected phenotype according to the patient’s *SMN2*_CN [57]. Lastly, variants in regulatory regions outside the gene could modify its expression, either increasing or decreasing the amount of SMN protein produced by each copy of *SMN2* as occurs with the modifier variants described within the gene. In fact, several targets of transcriptional regulation in the *SMN2* locus are under study [58,59].

Several discordant haploidentical SMA siblings have been described, presenting the same number of *SMN2* copies [60] and no differences by NGS analysis [9]. These cases are thought to be modulated by additional SMA genetic modifiers. Considering other possible SMA modifier genes, aside from *SMN2*, there are two candidates within the 5q13 region, *NAIP* (neuronal apoptosis inhibitory protein) and *SERF1A* (small EDRK-rich factor 1a). These genes have been found to be deleted in a proportion of patients, particularly with a more severe phenotype. However, results are inconclusive and it is more likely to be caused by a contiguity effect of the *SMN* deletion [61,62]. In addition, other factors outside the *SMN* gene locus may be involved in the *SMN2* response-activity and in the SMA phenotype’s definition. These factors are usually divided into *SMN*-dependent factors, which directly alter the amount of SMN protein, and *SMN*-independent factors playing a role in many different functions such as actin polymerization, cytoskeleton dynamics or neurogenesis [63]. For instance, *PLS3* (plastin 3) gene has been proposed to possibly modulate SMA disease progression in discordant SMA siblings as higher expression levels were found in lymphoblasts in sisters with milder phenotypes [64]. However, up to date, no DNA markers or modifiers that lie outside the SMA region have been validated in clinical settings [9]. Nevertheless, a thorough discussion of these modifiers can be found elsewhere [63,65]. A representative list of possible modifiers of SMA phenotype and their references is summarized in Table 2.

## 4. Evolving Therapies and the Importance of *SMN2*

In the SMA therapeutic background, there are three approved therapies by the FDA (Food and Drug Administration) and EMA (European Medicines Agency): nusinersen, risdiplam and onasemnogene abeparvovec-xioi (OA). Nusinersen and risdiplam agents are designed to bind specifically to *SMN2* pre-mRNA in order to promote exon 7 inclusion increasing the amount of functional SMN protein [76,77]. The remainder approved SMA treatment OA, consists of a gene replacement therapy that restores the expression of normal *SMN1* using a viral vector (AAV9) expressing *SMN1* [78]. While the *SMN2* endogenous target regions comprise splicing regulators and intronic regions, the *SMN1* transgene in the AAV9 is an *SMN1* cDNA lacking intronic or other regulatory elements. At first sight, the identification of *SMN2* copies, variants and structures would be of particular interest for the approved SMA treatments targeting *SMN2.* However, this information could be also useful for patients treated with OA. Indeed, the c.859G>C variant was relevant in the AVXS-101 trial as it was defined as an exclusion criteria, albeit the approval was given without limitation on the genetic background [78]. For example, in the context of presymptomatic detection, a neonate with 2 copies of *SMN2* carrying the c.859G>C variant will have a better evolution than typical cases with 2 *SMN2* copies without this modifier variant [39]. In view of the current progress in the worldwide implementation of neonatal screening, when a neonate is genetically diagnosed with SMA, it is recommended to perform not only *SMN2*_CN determination, but also a more complete *SMN2* characterization, including variants and if possible, structural changes [42]. While the influence of *SMN* hybrid genes and partial deletions on the response of the different therapies has not yet been addressed, it can be speculated that therapeutic efficacy of *SMN2* modulators may be affected depending on particular hybrid structures [10]. The NGS approach of the complete SMN2 genes is also useful to determine regions of the *SMN2* that are apparently highly conserved within the patients. For example, the ISSN1 sequence of intron 7, an interesting region because it is the target of the 18bp oligonucleotide nusinersen, appears to be so far identical in SMA patients [10], but further studies with a larger number of cases should be performed to confirm this observation.

Upon approvals, the availability of different therapies is complicating the decision-making for treatment choices. Besides monotherapy, several SMA patients are receiving combinatorial therapies starting with nusinersen and after OA or vice versa [79,80,81], and combination could also include risdiplam (individual reports). In principle, the mechanisms of action of these therapies do not interfere specifically with each other but are rather complementary. For example, any patient receiving gene therapy, will produce SMN protein autonomously and theoretically indefinitely as an episome in the nucleus of postmitotic cells and the endogenous *SMN2* could still be a possible target for *SMN2* modulators. However, the regulation and feedback that modulate SMN production by both mechanisms have not been elucidated yet. Recently, it has been reported that the overexpression of SMN protein by AAV9 has long-term neuronal toxic effects in a SMA mouse model [82]. Therefore, the putative overproduction of SMN with combinatorial therapies should also be cautiously considered in SMA patients, particularly in those who already received gene therapy. A recent consensus statement on gene replacement therapy for SMA does not recommend combinatorial therapy as part of routine care [83]. Further studies should be performed to determine when combinatorial therapies would be more effective than monotherapy. In the current scenario, combinatorial therapies may also include SMN-independent compounds, which are worth to be further investigated in SMA patients [84]. Interestingly, a recent study has showed promising results with the combinatorial use of an antisense oligonucleotide (ASO) mimicking nusinersen and an orally delivered histone deacetylase inhibitor (panobinostat) in SMA cell models. They have found that this compound increases the effects of the ASO on *SMN2* exon 7 inclusion enhancing the expression of *SMN2* [85]. As this is a constantly evolving field, regardless of the treatment received, all SMA cases may benefit from knowing their complete *SMN2* genotype to make better and tight correlations with each phenotype and more realistic outcomes after therapies.

## 5. Conclusions

**Figure 3:**
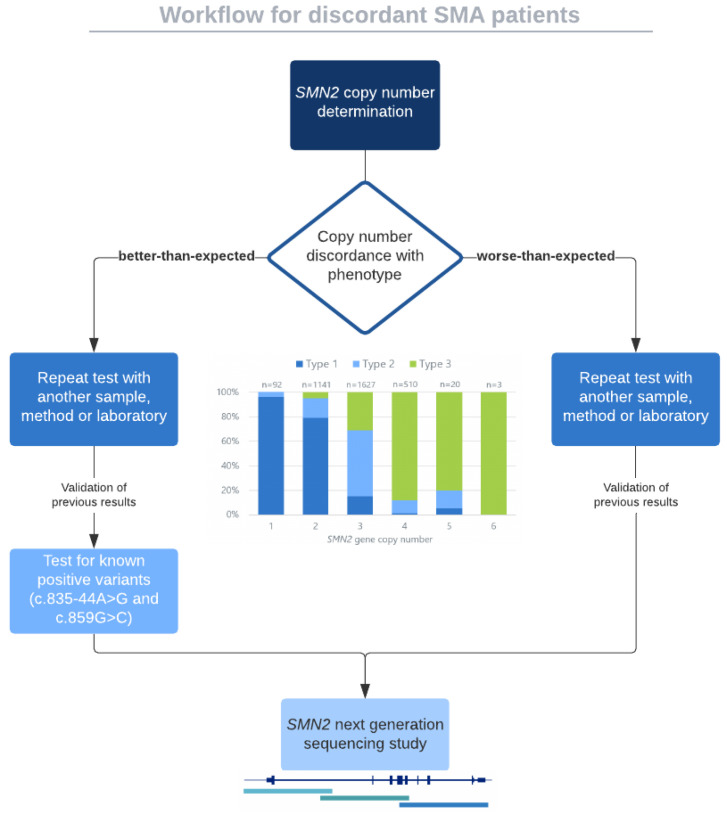
Workflow for discordant SMA symptomatic patients. Once *SMN2* is determined and discordance is found (according to the graphic in the center of the workflow based on the meta-analysis of 3459 cases from Calucho et al. (2018) [15]), a re-test should be performed with a new sample, another method or even another laboratory. If the discrepancy is confirmed, when the phenotype is better than expected, we may test for known and validated positive variants (c.859G>C and c.835-44A>G). If negative, we may continue with further NGS studies (as in [10]). In case that the phenotype is worse than expected, we should perform an NGS test in order to determine potential negative modifiers, hybrid structures or intragenic deletions that may explain the phenotype. Based on reference [42].

The present therapeutic scenario highlights the importance to genetically confirm SMA patients in order to make them eligible for treatment options. Although the vast majority of patients can be straightforwardly diagnosed, it is important to be aware of rare particular *SMN1* deletions or variants that may be critical to genetically confirm a given patient [56]. Once bi-allelic *SMN1* alterations are confirmed, *SMN2* enters the scene for better prognostic and phenotype characterizations. Discordant situations of genotype-phenotype correlation in SMA exist including when a phenotype of a given patient is better-than-expected according to *SMN2* copies (fewer copies, better phenotype) or conversely, when the phenotype is worse-than-expected according to *SMN2* copies (more copies, but more severe phenotype). These discordances may be due to biological or technical issues and all discordant cases should be retested considering a new sample, a different methodology and/or even another laboratory (Figure 3). Testing for positive known variants that may influence the amount of complete SMN should be performed once the copy number results are confirmed. NGS studies of the entire gene allow further characterization of the quality of *SMN2* copies. Better-than-expected discordances are usually explained by known positive variants in *SMN2*, but some cases still remain unexplained and further genetic investigations may unravel potential causes that explain the phenotype. In our experience, worse-than-expected cases are usually due to technical pitfalls (*SMN2*_CN overestimation), although negative modifiers are under validation studies. Partial deletions of *SMN2* may be masked if *SMN2* sequencing or quantitation is not carefully evaluated. Copy number results may be complemented with NGS of the entire *SMN2* genes to define their sequence and structure and detect further modifiers. Confirmation of copy number can also be achieved with NGS studies [10]. Finally, the therapeutic context of SMA is becoming more complex and expanded with several SMN-dependent or SMN-independent therapeutic approaches. Thus, combinatorial therapies are expected to be protocolized in the future, when more evidence about their efficacy is available [84]. All these advances should consider *SMN2* copies, variants and structures as part of the integral characterization of patients receiving expensive and sometimes lifelong therapies. The *SMN2* gene, as the main modifier of SMA phenotype, warrants a deeper study beyond the copy number determination. In the near future, either in the presymptomatic neonatal screening scenario or in already symptomatic patients, routine analysis may be adapted to currently detect rare (modifier) variants, single-nucleotide polymorphisms and structural variants of the SMN locus.

## Figures and Tables

**Table 1:** Complex characteristics of *SMN* genomic region and associated technical limitations. Left column shows the biological issues and the right column the technical difficulties associated with the analysis of all these complex issues.

Complex Biological Features of SMN Region	Technical Limitations
High homology between *SMN1* and *SMN2*	Difficulty in establishing if specific variants belong to *SMN1* or *SMN2*
Multiple copies of *SMN* genes	Inaccurate copy number determination
Partial deletions	Undetectable by routine analysis (exonic sequencing and MLPA)
*SMN2/SMN1* hybrid structures	
Unknown variants in deep intronic regions	

**Table 2:** Possible modifiers of spinal muscular atrophy. This is a representative list to show the different lines of investigation to determine factors that may modify the SMN function and SMA phenotype. Even though we include several factors, the *SMN2* gene (copy number, sequence and structure) is the only validated as a DNA marker. Based on references [15,63,64,65,66,67,68,69,70,71,72,73,74,75]. Arrow indicates increase (up) or decrease (down).

Modifier Type	Example	Effect	Reference
SMA locus	*SMN2* copies and variants	SMA types	Calucho et al. 2018 [15]
Splicing regulators	hnRNP-A1/Sam68	Exon 7 inclusion	Pedrotti et al. 2010 [66]
SMN degradation	*UBA1*	↑ Survival SMA mice	Powis et al. 2016 [67]
DNA methylation	*SLC23A2/NCOR2*	SMA types differences	Zheleznyakova et al. 2015 [68]
Actin polymerization	*PLS3*	Siblings differences	Oprea et al. 2008 [64]
Cytoskeleton dynamics	*ERK*	↑ Survival SMA mice	Branchu et al. 2013 [69]
Endocytosis regulators	*NCALD*	Ameliorates SMA	Riessland et al. 2017 [70]
Neurogenesis regulators	*PTEN*	↑ Survival SMA mice	Little et al. 2015 [71]
Axogenesis	*ZPR1*	↓ in SMA patients	Helmken et al. 2003 [72]
Apoptosis	Bcl2	↓ SMA motor neurons	Soler-Botija et al. 2003 [73]
Hormones/growth factors	Prolactin	↑ Survival SMA mice	Farooq et al. 2011 [74]
Environmental factors	Exercise	↑ Survival SMA mice	Grondard et al. 2005 [75]

## Data Availability

Not applicable
